# Differential hemodynamics between arteriovenous fistulas with or without intervention before successful use

**DOI:** 10.3389/fcvm.2022.1001267

**Published:** 2022-11-03

**Authors:** Hannah Northrup, Yong He, Ha Le, Scott A. Berceli, Alfred K. Cheung, Yan-Ting Shiu

**Affiliations:** ^1^Division of Nephrology and Hypertension, Department of Internal Medicine, University of Utah, Salt Lake City, UT, United States; ^2^Division of Vascular Surgery and Endovascular Therapy, University of Florida, Gainesville, FL, United States; ^3^Malcolm Randall Veterans Affairs Medical Center, Gainesville, FL, United States; ^4^Veterans Affairs Medical Center, Salt Lake City, UT, United States

**Keywords:** arteriovenous fistula, intervention, assisted maturation, vascular access, ESKD, hemodynamics

## Abstract

A significant number of arteriovenous fistulas (AVFs) fail to maturate for dialysis. Although interventions promote maturation, functional primary patency loss is higher for AVFs with interventions (assisted maturation) than AVFs without interventions (un-assisted maturation). Although blood flow-associated hemodynamics have long been proposed to affect AVF remodeling, the optimal hemodynamic parameters for un-assisted maturation are unclear. Additionally, AVF maturation progress is generally not investigated until 6 weeks after AVF creation, and the examination is focused on the AVF’s venous limb. In this exploratory study, patients (*n* = 6) underwent magnetic resonance imaging (MRI) at 1 day, 6 weeks, and 6 months after AVF creation surgery. Before successful use for hemodialysis, three AVFs required intervention and three did not. MRI of the AVFs were used to calculate lumen cross-sectional area (CSA) and perform computational fluid dynamics (CFD) to analyze hemodynamics, including velocity, wall shear stress (WSS), and vorticity. For the venous limb, the no-intervention group and intervention group had similar pre-surgery vein diameter and 1-day post-surgery venous CSA. However, the no-intervention group had statistically larger 1-day venous velocity (0.97 ± 0.67 m/s; mean ± SD), WSS (333 ± 336 dyne/cm^2^) and vorticity (1709 ± 1290 1/s) than the intervention group (velocity = 0.23 ± 0.10 m/s; WSS = 49 ± 40 dyne/cm^2^; vorticity = 493.1 ± 227 1/s) (*P* < 0.05). At 6 months, the no-intervention group had statistically larger venous CSA (43.5 ± 27.4 mm^2^) than the intervention group (15.1 ± 6.2 mm^2^) (*P* < 0.05). Regarding the arterial limb, no-intervention AVF arteries also had statistically larger 1-day velocity (1.17 ± 1.0 m/s), WSS (340 ± 423 dyne/cm^2^), vorticity (1787 ± 1694 1/s), and 6-month CSA (22.6 ± 22.7 mm^2^) than the intervention group (velocity = 0.64 ± 0.36 m/s; WSS = 104 ± 116 dyne/cm^2^, *P* < 0.05; vorticity = 867 ± 4551/s; CSA = 10.7 ± 6.0 mm^2^, *P* < 0.05). Larger venous velocity, WSS, and vorticity immediately after AVF creation surgery may be important for later lumen enlargement and AVF maturation, with the potential to be used as a tool to help diagnose poor AVF maturation earlier. However, future studies using a larger cohort are needed to validate this finding and determine cut off values, if any.

## Introduction

Arteriovenous fistulas (AVFs) are the preferred vascular access for chronic hemodialysis, with fewer complications (e.g., infection, thrombosis etc.) than alternative accesses (1, 2). However, up to 60% of AVFs fail to become useful for dialysis (3, 4). Although interventions, such as balloon angioplasty, can be used to achieve AVF maturation (assisted maturation), Lee et al. showed that functional primary patency loss within 2 years following AVF maturation is higher for AVFs with assisted maturation (82%) than AVFs that maturate without interventions (un-assisted maturation) (74%) (5).

Arteriovenous fistula maturation requires sufficient enlargement of the fistula vein lumen, which is thought to be induced by the increased blood flow due to directly shunting arterial blood to the vein and accompanying hemodynamic parameters (6). Current KDOQI guidelines recommend newly created AVFs to be evaluated, based on vessel diameter and blood flow, for maturation 4–6 weeks after creation (7). Patients requiring immediate dialysis must use a catheter in the interim, risking infection and morbidity associated with catheter use (8).

Despite extensive research over the past several decades, there is no effective therapy for promoting AVF maturation beyond surgical and endovascular interventions (9, 10). The lack of effective treatment is attributed to a poor understanding of the pathophysiology underlying AVF non-maturation (6, 11, 12). Computational fluid dynamics (CFD) studies can be used to understand the relationships between hemodynamics and AVF maturation. Hemodynamic parameters include fluid wall shear stress (WSS) and oscillatory shear index (OSI) on the wall (13), and vorticity and helicity in the lumen (14, 15). Arterial literature has shown that disturbed flow on the vessel wall, characterized by low WSS with high OSI, induces inflammatory, proliferative, and thrombotic responses (6). Disturbed flow in the lumen, characterized by eddies and spiral flow (6, 16), is associated with atherosclerotic lesion formation (16). It is not yet clear whether disturbed flow has the same effects on the vein or in the context of AVF remodeling.

Although there are several published CFD studies of AVFs (17), a disconnect remains between clinical and CFD research of AVFs. First, clinical research and standard care often follow the progression of AVF remodeling for several months after AVF creation, but CFD research usually focuses on a single time point soon after AVF creation (18–22), overlooking the temporal relationship between hemodynamics and AVF remodeling. Secondly, maturation as defined by the successful hemodialysis use of the AVF, is an outcome commonly used in clinical research (23–25), and yet no previous CFD reports considered with vs. without interventions before successful hemodialysis use as a clinical outcome. Third, the vein is the cannulation site for hemodialysis, and therefore, previous CFD research primarily focused on venous flow and remodeling and neglected the AVF artery. This study addresses these issues by characterizing venous and arterial hemodynamic parameters longitudinally for 6 months after AVF creation surgery, with the clinical outcome being with vs. without interventions before successful hemodialysis use.

Impaired AVF remodeling is often associated with stenosis (6). The juxta-anastomosis, the region up to 2–5 cm away from the anastomosis, is particularly susceptible to stenosis and thus requires interventions. Therefore, this region is the focus of this study. Here we present analysis from 18 CFD simulations of AVF blood flow performed using magnetic resonance imaging (MRI) scans of AVFs acquired at 1 day, 6 weeks, and 6 months after their creation. The time point of 1 day was chosen to obtain baseline values immediately following AVF creation surgery 6 weeks was chosen for the second scan because in many hospitals the standard of care is to assess AVF diameter and flow rate at this time (26). A total of 6 months was chosen because newly created AVFs are usually used for dialysis 6 months or later after creation. In addition, many AVFs fail to mature to adequately support dialysis within 6 months after AVF creation surgery (3). Using CFD simulation methods we analyzed lumen cross-sectional area (CSA), velocity, WSS, OSI, vorticity, and helicity in the artery and vein. In this exploratory study, we hypothesized that early hemodynamics (1 day after AVF creation) may be indicative of the need for intervention and that the hemodynamics of AVF vessels, throughout the first 6 months after AVF surgery, differ between the AVFs with vs. without interventions before their successful use for hemodialysis.

## Materials and methods

### Study patients and imaging

Patients were from the University of Utah, whose institutional review board approved the study protocol for this retrospective study of prospectively collected data. All recruited patients provided written informed consent. Patients received a one-stage, upper extremity AVF creation surgery and then underwent MRI approximately 1 day, 6 weeks, and 6 months after AVF creation surgery, as previously described (27). The average pre-operative diameter for the patients was 0.33 ± 0.10 cm.

Two-dimensional (2D) black-blood and cine-phase contrast scans were performed by contrast-free Siemens 3.0 Tesla MRI (Siemens Healthcare, Hoffman Estates, IL, USA) as previously described (27–29). The MRI acquisition protocol varied slightly from patient to patient in order to obtain the best quality scans as possible. A 2D black-blood turbo-spin echo/fast-spin echo with double inversion recovery preparation was used to obtain lumen geometry images. The imaging parameters were TE/TR (echo time/repetition time) of 8.8/915 ms, an echo train length of 9, pixel bandwidth of 250 Hz, and 0.5 mm × 0.5 mm × 2.0 mm (interpolated to 0.25 mm × 0.25 mm × 2.0 mm) slices. To identify the location for 2D cine-phase contrast imaging, 2D time of flight (TOF) was obtained with TE/TR of 7/25 ms, pixel bandwidth of 80 Hz and 0.3 mm × 0.3 mm × 2.0 mm slices. 2D cine-phase contrast was used to obtain blood flow rate with the following parameters: TE/TR of 3.6/25 ms, pixel bandwidth of 260 Hz and 0.7 mm × 0.7 mm × 3.0 mm slices, with the imaging plane perpendicular to the blood flow in a section of the vessel that was relatively straight. Velocity encoding values typically ranged from 60 to 220 cm/s based on maximal velocity. The pulse obtained from a finger oximeter was used for gating during the phase-contrast scan. The day 1 scan was performed to obtain post-surgery baseline values following AVF creation surgery. The second scan was performed when patients underwent their standard-of-care ultrasound assessment at 6 weeks after AVF creation. Newly created AVFs are usually used for dialysis 6 months or later after creation, and thus the third was performed at 6 months.

Patients were chosen based on several criteria. First, patients had similar pre-surgery vein diameters. Second, all three MRI scans for each patient were available with good quality for CFD analysis. Third, these AVFs were successfully used for three consecutive hemodialysis sessions. Patients whose AVFs were successfully used for three consecutive hemodialysis sessions without prior interventions were put in the no-intervention group (*n* = 3) (2 upperarm, 1 forearm). Patients whose AVFs required intervention (angioplasty at 9 weeks after AVF creation) before successful use of the AVF for three consecutive hemodialysis sessions were put in the intervention group (*n* = 3) (1 upperarm, 2 forearm). Table 1 shows the patient characteristics and pre-surgery vein diameters. In total, AVFs from 6 patients were analyzed at 1 day, 6 weeks and 6 months for a total of 18 MRI scans and 18 patient-specific CFD simulations.

**TABLE 1:** Individual patient characteristics.

Variable	No-intervention			Required intervention		
Age, year	58	52	50	58	34	62
Sex	Female	Female	Male	Male	Male	Male
Race	White, Caucasian	White, Caucasian	Black, African American, African	Black, African American, African	White, Caucasian	White, Caucasian
On dialysis at time of surgery	No	Yes	No	Yes	Yes	Yes
Diabetes	Yes	No	No	Yes	Yes	Yes
Hypertension	Yes	Yes	Yes	Yes	Yes	Yes
History of cerebrovascular disease	Yes	No	No	No	No	No
AVF type	Forearm	Upper arm	Upper arm	Forearm	Upper arm	Forearm
Pre-surgery vein diameter (cm)	0.15	0.53	0.35	0.12	0.29	0.27

### Computational fluid dynamics simulations

**FIGURE 1:**
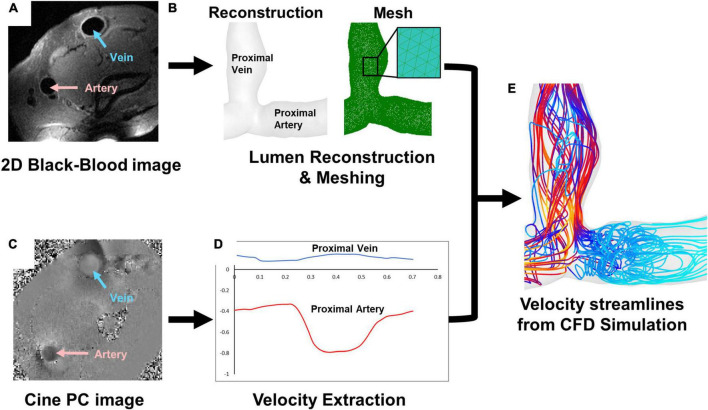
Magnetic resonance imaging (MRI) and simulation pipeline. **(A)** 2D black-blood MRI scan for **(B)** lumen reconstruction and mesh. **(C)** Cine-phase contrast **(PC)** scan for **(D)** velocity extraction, which shows an example of velocity in the proximal vein **(blue)** and proximal artery **(red)**. **(E)** An example of velocity streamlines from CFD simulation.

Computational fluid dynamics simulations were performed as previously described (Figures 1A–E) (27). 2D black-blood MRI scans were used in Amira (Thermo Fisher Scientific, Waltham, MA, USA) to reconstruct the 3D lumen geometries of each AVF (Figures 1A,B). With the disordered flow of the AVF, MRI near the anastomosis can sometimes loose signal quality. To compensate for this and as an additional check on the accuracy of the reconstructions, the 3D geometries were qualitatively compared with maximum intensity projections created from time-of-flight MRI images (Supplementary Figure 1). To prevent interference at the inlets and outlets and ensure fully developed fluid flow in the region of interest, flow extensions were added to all openings of the lumen geometry using VMTK with an extension length 10 times that of the lumen diameter (available at)^1^ (Supplementary Figure 2). The effect of different lengths (and/or tapering) of flow extensions on the region of interest is outside of the present study’s scope and can be considered in future studies. The data collected for analysis did not include the flow extensions. Meshing was performed in ICEM CFD 2020 R1 (Ansys, Inc., Canonsburg, PA, USA). Based on previous mesh-dependence studies (27, 30), approximately 1.5 million tetrahedra with 4 prism layers were used (Figure 1B). Blood flow was extracted using the cine-phase contrast MRI scans in Segment (available at medviso.com) (Figure 1C) and averaged over the inlet CSA. The resulting flow data was used to calculate the velocity at the boundary conditions for each time step, resulting in a pulsatile plug velocity profile specific to each vessel (Figure 1D).

Simulations were performed in Ansys Fluent 2020 R1, assuming a laminar flow with a rigid wall. These 6 patients’ mean Reynolds numbers were 804 ± 485, within the laminar flow range. Blood was prescribed as pulsatile, Newtonian with a viscosity of 0.0035 kg/m⋅s, and incompressible with a density of 1050 kg/m^3^. The inlets and outlets included the proximal vein (PV), proximal artery (PA), and distal artery. One of the lumen ends was set up as a pressure outlet with 0 pressure, and the other two ends were inlets using the extracted velocity described above (Figure 1D). The solution methods followed the Semi Implicit Method for Pressure Linked Equations (SIMPLE) scheme with spatial discretization set to least squares cell based for gradient, second order for pressure, and second order upwind for momentum, which were used in our previous publications (15, 27, 30–32). The transient formulation was set to second order implicit, and residuals were set to 1e-5 for continuity and x, z, and z velocity for the convergence criteria. The time step was set to 1/10th of the velocity step size used in phase-contrast MRI or approximately 0.0025 s which has been shown to adequately characterize flow in previous studies (32). Simulations were run for 120 time steps plus one cardiac cycle. Data was analyzed from the last cardiac cycle.

### Post-computational fluid dynamics simulation processing

Lumen centerlines starting from the anastomosis to the ends of the PV and PA were created in VMTK, each point 1 mm apart. The centerlines were used in MATLAB (MathWorks, Natick, MA, USA) to calculate the vector normal to each centerline point. The centerline and normal vectors were used to create slices in Tecplot 360 (Tecplot Inc., Bellevue, WA, USA). The CSA of each slice was calculated in Tecplot. Velocity was calculated as described in Equation 1 where *u_*x*_, u_*y*_*, and *u*_*z*_ are components of the velocity vector *u.*


(1) $${\left(u_{x}^{2}+u_{y}^{2}+u_{z}^{2}\right)}^{\frac{1}{2}}$$

WSS was calculated as described in Equation 2 where τ_*w,x*_, τ_*w,y*_, and τ_*w,z*_ are the components of WSS_*w*_.


(2) $$W\,S\,S={\left(\tau_{w,x}^{2}+\tau_{w,y}^{2}+\tau_{w,z}^{2}\right)}^{\frac{1}{2}}$$

Oscillatory shear index was calculated as described in Equation 3 where *T* is the period of the cardiac cycle.


(3) $$O\,S\,I=0.5\,\left(1-\frac{\left|\int_{o}^{T}\tau_{w}\,d\,t\right|}{\int_{o}^{T}\left|\tau_{w}\right|\,d\,t}\right)$$

Vorticity was calculated as described in equation 4.


(4) V⁢o⁢r⁢t⁢i⁢c⁢i⁢t⁢y⁢(Ω)=∇×u

Relative helicity was calculated as described in Equation 5 and the absolute value of relative helicity reported as the magnitude of relative helicity.


(5) $$R\,e\,l\,a\,t\,i\,v\,e\,h\,e\,l\,i\,c\,i\,t\,y=\frac{u\,\Omega }{\left|u\right|\,\left|\Omega \right|}$$

All hemodynamic parameters (velocity, WSS, OSI, vorticity, and relative helicity magnitude) were averaged over a cardiac cycle. Data were taken from slices in the juxta-anastomosis (0–2 cm away from the anastomosis) in both the PA and PV.

### Statistics

Statistical analyses were performed using GraphPad Prism (GraphPad Software, San Diego, CA, USA). For CSA and hemodynamic analysis, data were taken from each slice of the AVF for a total of 20 data points per AVF (1 data point for every slice located every 1 mm along the centerline). For rate of change the data were averaged to result in one data point per AVF. D’Agostino-Pearson tests were performed to determine normality as recommended by GraphPad Prism. To assess significance a Kruskal–Wallis with Dunn’s multiple comparison test was performed. *P*-values were adjusted automatically by Graphpad Prism. Unpaired *t*-tests were used for pre-surgery ultrasound-measured diameter. All box and whisker graphs show the 25th to 75th percentiles within the box, with the whiskers extending to the minimum and maximum values and the line in the middle of the box as the median. Differences were considered significant if *P* < 0.05.

## Results

### Lumen size and flow

**FIGURE 2:**
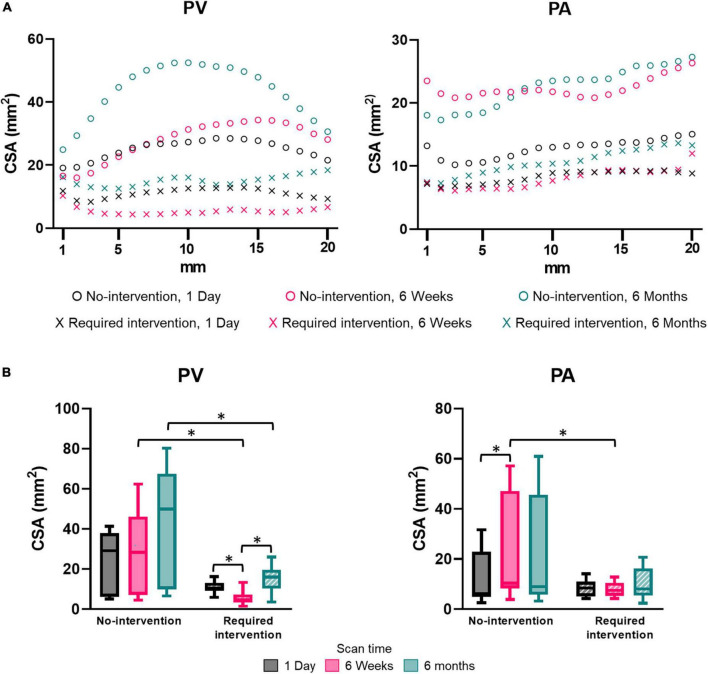
Cross-sectional area (CSA) of the proximal vein (PV) and proximal artery (PA). **(A)** CSA by distance away from the anastomosis at an increment of 1 mm. Each marker shows the average of three patients. **(B)** CSA for the “no intervention” and “required intervention” groups at 1 day, 6 weeks, and 6 months. CSA was averaged over the distance of 20 mm starting from anastomosis, as seen in **(A)**. The box and whisker graph shows the 25th to 75th percentiles within the box, with the line in the middle of the box as the median and the whiskers extending to the minimum and maximum values. *P < 0.05.

Pre-surgery forearm and upper-arm vein diameters measured by ultrasound in the no-intervention (0.21 ± 0.07 cm and 0.36 ± 0.13 cm, respectively) and intervention (0.21 ± 0.06 cm and 0.30 ± 0.05 cm, respectively) groups were similar (*P* > 0.05). Post-surgery venous lumen size was expressed as CSA. CSA was not uniform along the vascular axis for both groups and generally increased when farther away from the anastomosis (Figure 2A). The PV CSA of the no-intervention group increased from 1 day to 6 weeks to 6 months; in contrast, the PV CSA of the intervention group decreased from 1 day to 6 weeks (*P* < 0.0001) and, upon receiving intervention at approximately 9 weeks, increased from 6 weeks to 6 months (*P* < 0.0001) (Figure 2B and Table 2). The PV CSA in the no-intervention group was approximately twofold greater at 1 day (*P* = 0.12), fivefold greater at 6 weeks (*P* < 0.0001), and threefold greater at 6 months (*P* = 0.0005) than the PV CSA in the intervention group.

**TABLE 2:** Results of cross-sectional area (CSA), velocity, wall shear stress (WSS), oscillatory shear index (OSI), vorticity, and relative helicity magnitude.

		Proximal vein		Proximal artery	
		No intervention	Intervention	No intervention	Intervention
CSA (mm^2^)	1 day	25.0 ± 14.4	11.2 ± 2.7	12.8 ± 10.6	8.3 ± 3.4
	6 weeks	27.9 ± 19.7	5.5 ± 2.8	22.4 ± 19.5	8.0 ± 2.8
	6 months	43.5 ± 27.4	15.1 ± 6.2	22.6 ± 22.7	10.7 ± 6.0
Velocity (m/s)	1 day	0.97 ± 0.67	0.23 ± 0.10	1.17 ± 1.05	0.64 ± 0.36
	6 weeks	0.80 ± 0.22	0.97 ± 0.62	0.96 ± 0.43	0.61 ± 0.38
	6 months	0.49 ± 0.12	0.71 ± 0.59	0.73 ± 0.71	0.45 ± 0.23
WSS (dyne/cm^2^)	1 day	333 ± 336	49 ± 40	340 ± 423	104 ± 116
	6 weeks	193 ± 123	365 ± 376	172 ± 184	106 ± 91
	6 months	108 ± 64	204 ± 222	158 ± 240	90 ± 96
OSI	1 day	0.046 ± 0.035	0.028 ± 0.026	0.021 ± 0.040	0.001 ± 0.004
	6 weeks	0.081 ± 0.044	0.015 ± 0.022	0.011 ± 0.030	0.010 ± 0.019
	6 months	0.070 ± 0.046	0.046 ± 0.044	0.043 ± 0.052	0.054 ± 0.070
Vorticity (1/s)	1 day	1709 ± 1290	493.1 ± 227	1787 ± 1694	867 ± 455
	6 weeks	1208 ± 465	1925 ± 1290	1114 ± 682	895 ± 552
	6 months	709 ± 245	1044 ± 665	1073 ± 1201	659 ± 381
Relative helicity magnitude	1 day	0.15 ± 0.07	0.16 ± 0.06	0.10 ± 0.07	0.08 ± 0.07
	6 weeks	0.09 ± 0.07	0.14 ± 0.11	0.05 ± 0.04	0.06 ± 0.07
	6 months	0.10 ± 0.09	0.18 ± 0.16	0.04 ± 0.04	0.07 ± 0.05

Data presented as mean ± SD.

The PA CSA was larger in the no-intervention group than the intervention group at each time, though only significantly at 6 weeks (*P* = 0.0004) (Figure 2 and Table 2). The PA CSA in the no-intervention group increased from 1 day to 6 weeks (*P* = 0.0026) and was similar at 6 weeks and 6 months. The PA CSA in the intervention group was similar in all three time points and was not affected by the intervention performed at the venous side.

### Velocity

**FIGURE 3:**
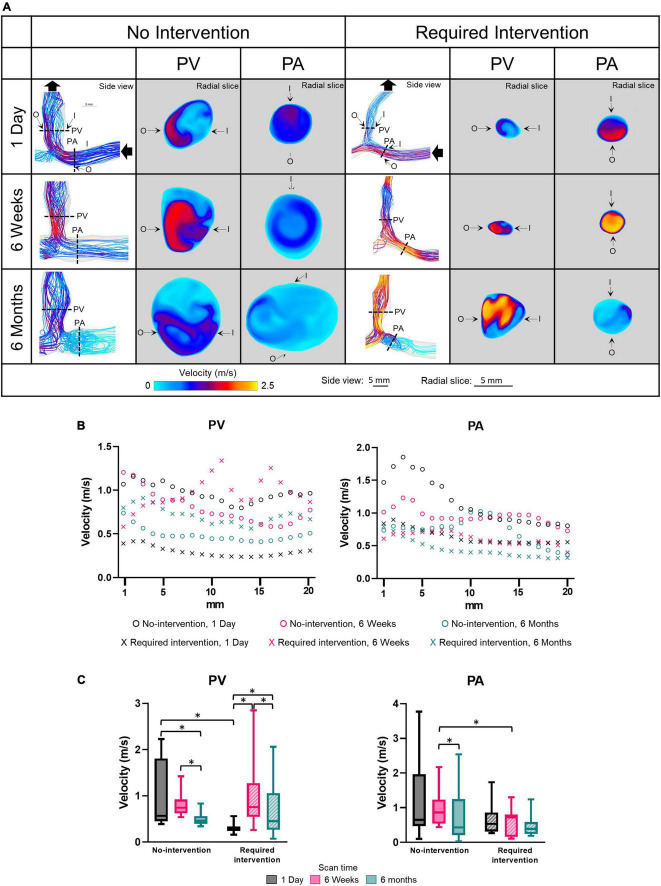
Velocity of the proximal vein (PV) and proximal artery (PA). **(A)** Velocity color maps of the side view and radial slices of representative arteriovenous fistulas at systole. Solid black arrows denote the flow direction. Dotted lines in the side view color maps show the location of radial slices, approximately 5 mm from the anastomosis. O and I denote the locations of outer and inner walls, respectively. **(B,C)** Show velocity for the “no intervention” and “required intervention” groups at 1 day, 6 weeks, and 6 months, as explained in Figure 2. *P < 0.05.

Side view images of velocity streamlines of all patients at all three time points are shown in Supplementary Figure 3. Figure 3 displays the representative post-AVF creation surgery velocity as velocity-magnitude color coated streamlines and radial slices of contour plots of velocity magnitudes of one no-intervention patient and one required intervention patient (Figure 3A), velocity magnitude along the vascular axis at 1-mm increment for 2 cm (Figure 3B) and averaged over 2 cm (Figure 3C and Table 2). Non-disturbed laminar flow has parallel streamlines, with the highest and lowest velocity in the lumen center and at the wall, respectively. The streamline plots show that both groups had eddies and spiral flows, and the radial slices show that the highest velocity was not in the lumen center, both features of disturbed flow.

The velocity magnitude was not uniform along the axis for both groups in either PV or PA and, in general, decreased farther away from the anastomosis. In the no-intervention group, the PV and PA velocities decreased with time. In contrast, the PV velocity in the intervention group increased from 1 day to 6 weeks (*P* < 0.0001) and, upon receiving the intervention at 9 weeks, decreased from 6 weeks to 6 months (*P* < 0.001). The PA velocity in the intervention group was similar in all three time points.

Proximal vein velocity was fourfold greater in the no-intervention group (0.97 ± 0.67 m/s) than in the intervention group (0.23 ± 0.10 m/s) (*P* < 0.0001) at 1 day. However, PV velocity was not significantly different between these 2 groups at 6 weeks or 6 months. As mentioned above, PV CSA became significantly greater in the no-intervention group than in the intervention group at 6 weeks and 6 months. Thus, for the vein, the bigger the velocity at 1 day, the bigger the CSA at 6 weeks and 6 months.

### Wall shear stress

**FIGURE 4:**
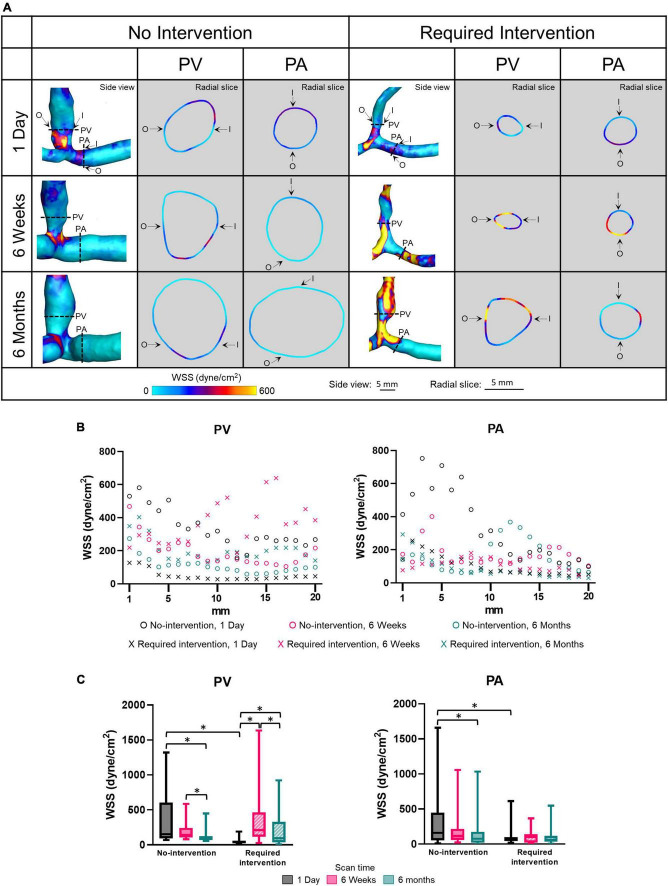
Wall shear stress (WSS) of the proximal vein (PV) and proximal artery (PA). **(A)** The side view and radial slices of WSS color maps of representative arteriovenous fistulas at systole. Dotted lines in the side view color maps show the location of radial slices, approximately 5 mm from the anastomosis. O and I denote the locations of outer and inner walls, respectively. **(B,C)** Show WSS for the “no intervention” and “required intervention” groups at 1 day, 6 weeks, and 6 months, as explained in Figure 2. ^*^P < 0.05.

Wall shear stress is the tangential force on the endothelial surface of the blood vessel due to the flowing blood. Side view images of WSS of all patients at all three time points are shown in Supplementary Figure 4. Figure 4 displays WSS magnitude as side views and radial slices of contour plots of one no-intervention patient and one required intervention patient (Figure 4A), along the vascular axis at 1-mm increment for 2 cm (Figure 4B) and averaged over 2 cm (Figure 4C and Table 2). WSS is 10 to 25 dyne/cm^2^ in normal arteries and 1 to 6 dyne/cm^2^ in normal veins (33) and is usually uniform along the circumference. WSS in the AVF’s PA and PV, regardless of groups, was drastically higher than the normal values and was not uniform along the circumference. The side view contour plots show that the highest WSS was localized to the anastomosis in the no-intervention group, but was more diffusive in the intervention group, distributing throughout a longer segment of the AVFs (Figure 4).

The WSS magnitude was not uniform along the vascular axis for both groups in either PV or PA and, in general, decreased when farther away from the anastomosis. In the no-intervention group, WSS in the PV and PA decreased from 1 day to 6 months (PV: *P* = 0.0006; PA: *P* = 0.0055). In contrast, the PV WSS in the intervention group increased from 1 day to 6 weeks (*P* < 0.0001) and, after intervention at approximately 9 weeks, decreased from 6 weeks to 6 months (*P* = 0.0017). The PA WSS in the intervention group was similar in all three time points.

Proximal vein WSS was sevenfold greater in the no-intervention group (333 ± 336 dyne/cm^2^) than in the intervention group (49 ± 40 dyne/cm^2^) (*P* < 0.0001) at 1 day; however, it was not significantly different between the 2 groups at 6 weeks or 6 months. Taken together with the PV CSA time course, the bigger the WSS at 1 day, the bigger the CSA at 6 weeks and 6 months.

### Oscillatory shear index

**FIGURE 5:**
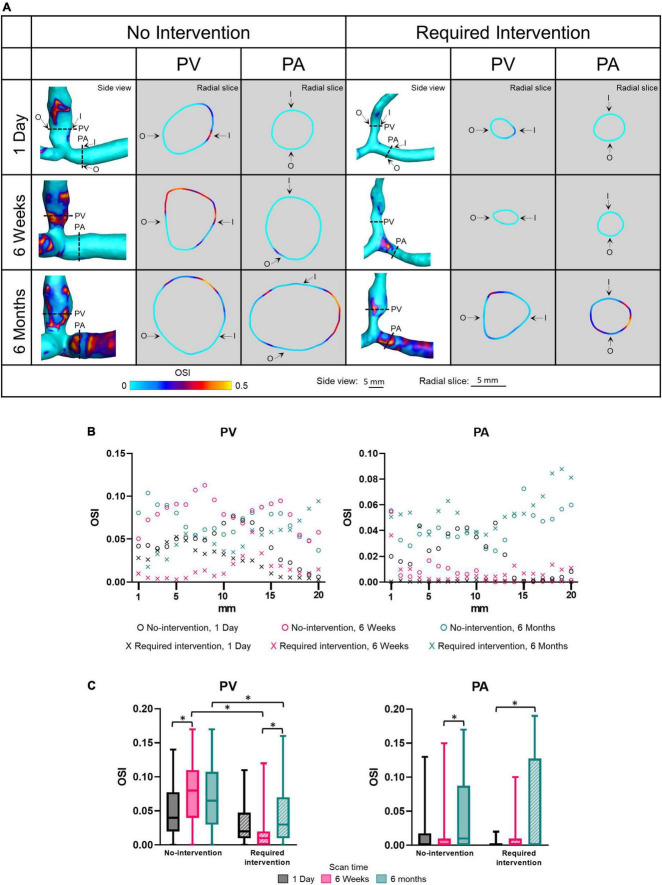
Oscillatory shear index (OSI) of proximal vein (PV) and proximal artery (PA). **(A)** The side view and radial slices of OSI color maps of representative arteriovenous fistulas. Dotted lines in the side view color maps show the location of radial slices, approximately 5 mm from the anastomosis. O and I denote the locations of outer and inner walls, respectively. **(B,C)** Show OSI for the “no intervention” and “required intervention” groups at 1 day, 6 weeks, and 6 months, as explained in Figure 2. ^*^P < 0.05.

The OSI describes the extent to which WSS changes its direction over a cardiac cycle; it ranges from 0 (i.e., no change) to 0.5 (flow reversal at half of the cardiac cycle). Side view images of OSI of all patients at all three time points are shown in Supplementary Figure 5. Figure 5 displays OSI as side views and radial slides of contour plots of one no-intervention patient and one required intervention patient (Figure 5A), along the vascular axis at 1-mm increment for 2 cm (Figure 5B) and averaged over 2 cm (Figure 5C and Table 2). Figures 5A,B show that OSI was heterogeneous throughout AVFs. In general, OSI increased with time in both groups and both artery and vein. Further, at 6 weeks and 6 months, the no-intervention group had significantly higher OSI than the intervention group in the PV. However, all values were small (see the third paragraph in “Discussion”).

### Vorticity

**FIGURE 6:**
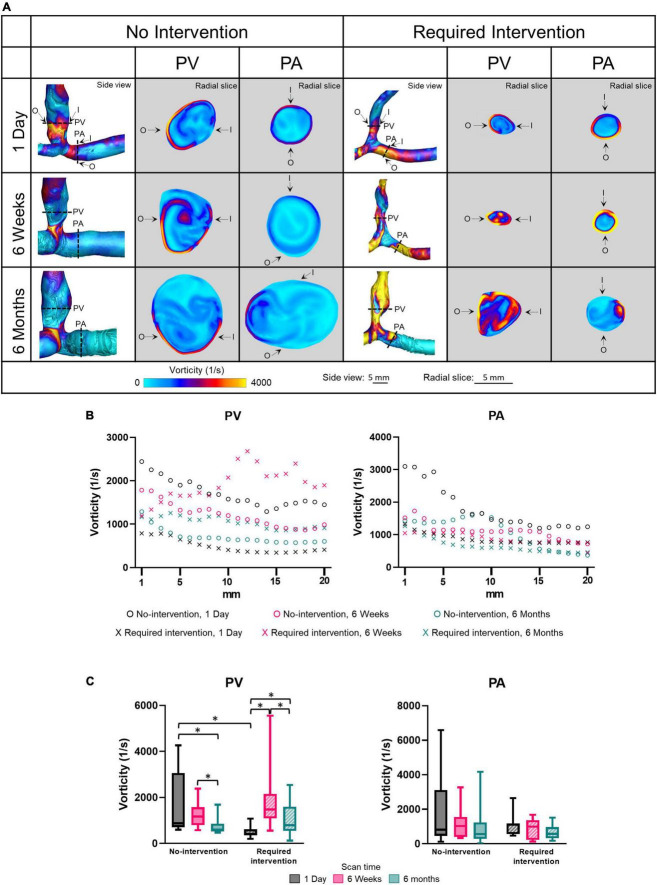
Vorticity of the proximal vein (PV) and proximal artery (PA). **(A)** The side view and radial slices of vorticity color maps of representative arteriovenous fistulas at systole. Dotted lines in the side view color maps show the location of radial slices, approximately 5 mm from the anastomosis. O and I denote the locations of outer and inner walls, respectively. **(B,C)** Show vorticity for the “no intervention” and “required intervention” groups at 1 day, 6 weeks, and 6 months, as explained in Figure 2. ^*^P < 0.05.

Vorticity quantifies the rotation of fluid flow in the lumen. Side view images of vorticity of all patients at all three time points are shown in Supplementary Figure 6. Figure 6 displays vorticity as side views and radial slices of contour plots of one no-intervention patient and one required intervention patient (Figure 6A), along the vascular axis at 1-mm increment for 2 cm (Figure 6B) and averaged over 2 cm (Figure 6C and Table 2). The side-view contour plots show high vorticity throughout the AVFs, and the radial slices show that the highest vorticity tended to locate near the wall in both groups at all three time points.

Vorticity was not uniform along the vascular axis for both groups in either PV or PA. The no-intervention group’s PV vorticity decreased from 1 day to 6 months (*P* < 0.0001). In contrast, the PV vorticity in the intervention group increased from 1 day to 6 weeks (*P* < 0.0001) and, after intervention at approximately 9 weeks, decreased from 6 weeks to 6 months (*P* < 0.0001). The PA vorticity in both groups was similar in all three time points.

Proximal vein vorticity was approximately 3.5-fold greater in the no-intervention group (1709 ± 1290 1/s) than in the intervention group (493.1 ± 227 1/s) (*P* < 0.0001) at 1 day; it was not significantly different between the 2 groups at 6 weeks or 6 months. Taken together with the PV CSA time course, the bigger the vorticity at 1 day, the bigger the CSA at 6 weeks and 6 months.

### Relative helicity magnitude

**FIGURE 7:**
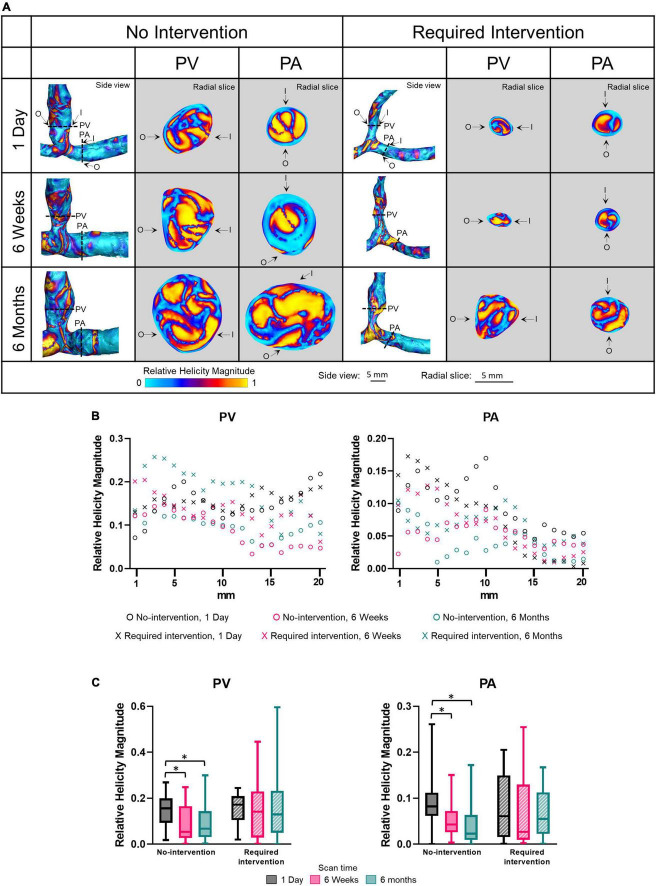
Relative helicity magnitude of the proximal vein (PV) and proximal artery (PA). **(A)** The side view and radial slices of relative helicity magnitude color maps of representative arteriovenous fistulas at systole. Dotted lines in the side view color maps show the location of radial slices, approximately 5 mm from the anastomosis. O and I denote the locations of outer and inner walls, respectively. **(B,C)** Show relative helicity magnitude for the “no intervention” and “required intervention” groups at 1 day, 6 weeks, and 6 months, as explained in Figure 2. ^*^P < 0.05.

Relative helicity quantifies the angle between the vorticity and velocity vectors. The relative helicity magnitude ranges from a value of 1 (i.e., complete alignment between the vorticity and velocity vectors) and 0 (the vorticity vector is perpendicular to the velocity vector). Side view images of relative helicity magnitude of all patients at all three time points are shown in Supplementary Figure 7. Figure 7 displays relative helicity magnitude as side views and radial slices of contour plots of one no-intervention patient and one required intervention patient (Figure 7A), along the vascular lumen axis at 1-mm increment for 2 cm (Figure 7B) and averaged over 2 cm (Figure 7C and Table 2). Figures 7A,B show that the relative helicity magnitudes were heterogeneous throughout AVFs. The relative helicity magnitude decreased in both the PV and PA over time in the no-intervention group (PV: 1 day vs. 6 weeks *P* = 0.0003, 1 day vs. 6 months *P* = 0.0038; PA: 1 day vs. 6 weeks *P* = 0.023, 1 day vs. 6 months *P* < 0.0001) but remained similar over time in the intervention group.

### Rate of change

**FIGURE 8:**
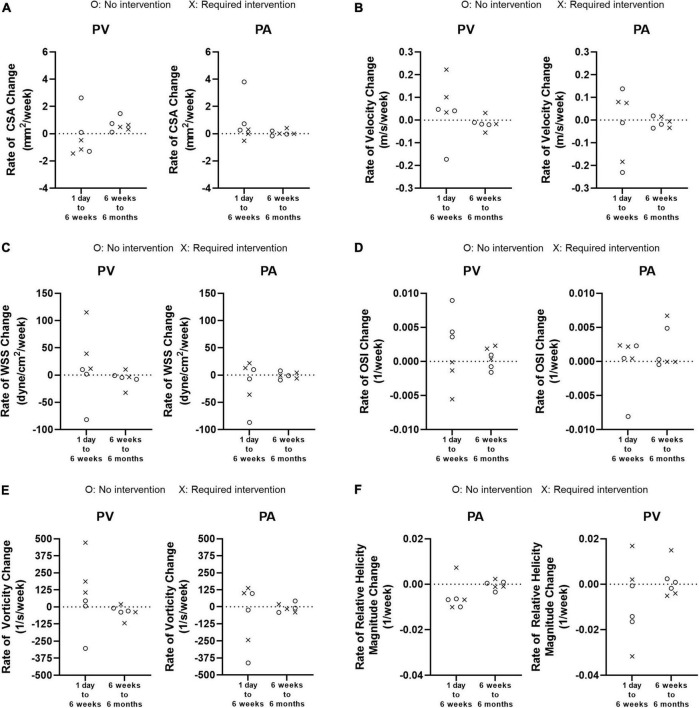
Weekly rate of change from 1 day to 6 weeks and from 6 weeks to 6 months. **(A)** Cross-sectional area (CSA). **(B)** Velocity. **(C)** Wall shear stress (WSS). **(D)** Oscillatory shear index (OSI). **(E)** Vorticity. **(F)** Relative Helicity Magnitude.

Figure 8 shows how quickly each parameter changed between 1 day and 6 weeks (the initial phase) and between 6 weeks and 6 months (the later phase). There was no significant difference for either PA or PV between the two groups for any parameter. However, when the two groups were combined, the rate of change for every parameter trended larger (either more positive or negative) in the initial phase than in the later phase, for both the PA and PV.

## Discussion

Although hemodynamics have long been proposed to influence AVF remodeling, it is unknown whether hemodynamic parameters are different in AVFs that maturate without vs. with intervention. Results in the present study have advanced our knowledge in this regard, laying the groundwork for future studies to investigate early hemodynamics in association with AVF maturation and intervention. Compared to the intervention group, the no-intervention group had higher velocity, WSS, and vorticity at 1 day and larger CSA at 6 weeks and 6 months in AVF veins, even though these two groups had similar pre-surgery vein diameter and their 1-day post-surgery venous lumen area were not statistically different. Thus, larger velocity, WSS, and vorticity immediately after AVF creation surgery may be important for later lumen enlargement in AVF veins. However, future studies with a larger cohort are needed to validate these findings.

A critical hemodynamic change triggering vascular remodeling post-AVF creation is WSS. Increased blood flow after AVF creation has long been hypothesized to promote an adaptive response in the AVF in which the luminal diameter increases to reduce WSS back to the pre-AVF level (34). We found that AVF creation drastically increased venous WSS in both groups from 1 to 5 dyne/cm^2^ in normal veins (33). However, 1-day venous WSS was significantly higher in the no-intervention group than in the intervention group. Further, while venous CSA in the no-intervention group increased from 1 day to 6 weeks, venous CSA in the intervention group decreased from 1 day to 6 weeks, leading to the required intervention. Our finding suggests the presence of a critical WSS threshold for lumen enlargement. Future studies with a larger patient number will be needed to confirm this concept and identify such a WSS threshold.

Oscillatory flow often occurs in regions prone to atherosclerosis and arterial neointimal hyperplasia (6), and an OSI at or above 0.2 has been used as a marker for associated risk of atherosclerosis (35). All OSI levels in the present study were below this threshold and may not be high enough to influence AVF remodeling. However, high levels of OSI can be seen in the color maps (Figure 5A). Thus, increased spatial resolution may be required to analyze OSI in the context of AVF remodeling. Additionally, in the arterial disease literature, high OSI in conjunction with low WSS has been hypothesized to induce hyperplasia and stenosis (36, 37). In our study, higher OSI was accompanied by higher WSS, probably due to the more disturbed flow under a larger flow velocity. It is possible that the benefit of higher WSS is more dominant than higher OSI in driving AVF remodeling. This study focuses on time averaged WSS and OSI because so far they are the most common WSS based metrics in AVF (37) and other vascular literature, such as in atherosclerosis (35). Other WSS based metrics, such as transverse WSS and relative residence time, may shed additional light on the relationship between hemodynamics and AVF remodeling and can be considered in future research.

Wall shear stress and OSI have received the most attention in investigating the hemodynamic regulation of vascular remodeling (37). These two parameters only describe the near-wall flow. To investigate whether luminal hemodynamic parameters differed between the no-intervention and intervention groups, we considered vorticity and relative helicity, which have been gaining attention because they can affect circulating blood cells and platelets and, subsequently, vascular walls. In arterial circulation, vortical and helical flow patterns have been associated with collagen-based vessel remodeling, inflammation, and increased vascular stiffness (14). In the present study, venous vorticity was significantly greater in the no-intervention group than in the intervention group at 1 day but was similar between both groups at 6 weeks and 6 months. Helicity was similar between both groups at all three time points. Thus, for the vein, the level of increase in vorticity, but not helicity, immediately after AVF creation surgery appears to be important for lumen enlargement.

Relatively little is known about arterial remodeling after AVF creation in patients, even though pre-surgery arterial diameter is associated with unassisted AVF maturation (38), and pre-surgery arterial flow-mediated dilation and nitroglycerin-mediated dilation are associated with 6-week AVF blood flow and diameter (39). In the present study, the arterial lumen area was larger in the no-intervention group than the intervention group at each time point, though only significantly at 6 weeks. One-day WSS and vorticity in the artery were larger in the no-intervention group than in the intervention group, though not significantly. More studies are needed to investigate the role of arterial remodeling in AVF maturation.

KDOQI clinical practice guidelines recommend that AVFs be evaluated for maturation 4–6 weeks after creation. The Hemodialysis Fistula Maturation Consortium study reported that ultrasound measurements of AVF flow and diameters at 1 day, 2 weeks, and 6 weeks predicted clinical maturation in a statistically significant manner (40). Thus, AVF remodeling within 6 weeks after creation is critical. Our study added to the literature by showing that the rate of change in lumen area and hemodynamic factors were larger between 1 day and 6 weeks than between 6 weeks and 6 months. We also showed that larger WSS and vorticity at 1 day were critical in promoting lumen enlargement. The trajectory toward AVF clinical maturation as predicted by ultrasound at 1 day, 2 weeks, and 6 weeks was similar (40). Studies investigating additional early time points, before 6 weeks, could help elucidate hemodynamic-mediated mechanisms of AVF maturation. For example, MRI-based CFD from scans taken each week during the first 6 weeks, could reveal how AVF hemodynamics and lumen area change week by week. However, weekly MRI scans would put burden on patients and thus may be more conducive to animal studies such as we have presented elsewhere (15, 30–32, 41).

The significantly decreased venous CSA from 1 day to 6 weeks in the intervention group suggests the presence of stenosis and may have caused the velocity, WSS, and vorticity to increase from 1 day to 6 weeks. Although WSS at 1 day in the no-intervention group was similar to WSS at 6 weeks in the intervention group, the former occurred immediately after AVF creation and the latter due to stenosis. Thus, the increase in venous CSA in the no-intervention group from 1 day to 6 weeks to 6 months may be driven by flow and WSS, whereas the higher CSA at 6 months than 6 weeks in the intervention group due to angioplasty.

While CFD-based longitudinal studies are rare in literature (27, 42, 43), other longitudinal studies of human AVFs investigated diameter and flow by ultrasound. In general, their temporal changes in flow and diameter follow a similar trend as the CSA from the no-intervention group in the present study. In the multicenter Hemodialysis Fistula Maturation study with 602 patients undergoing AVF creation surgery in the United States, ultrasound-measured diameter and blood flow was obtained at 1 day, 2 weeks, and 6 weeks after AVF creation surgery. Flow rate increased from 1 day to 6 weeks and were predictive at each time point of AVF maturation. Increase in diameter and flow rate over time were also found in two other small single center studies in Eurasia [*n* = 18, Lomonte et al. (44); and *n* = 31, Seyahi et al. (45)]. In a single-center study in China, ultrasound was performed to 132 patients at 1, 14, 28, 42, 56, 70, and 84 days after AVF creation surgery. They found that in both groups, matured vs. failed fistulas, although the blood flow and vascular diameters increased over time, blood flow in the failure group was significantly lower than that in matured; however, interestingly, diameter was not significantly different at most time points (46).

This study has several strengths. It offers detailed longitudinal hemodynamics of patient AVFs from 1 day to 6 months after AVF creation, which are rare in the literature (27, 42, 43). It combines clinical and computational data, reporting for the first time the importance of early WSS in driving lumen enlargement for unassisted maturation. Our study utilizes MRI instead of ultrasound. Although ultrasound is already standard of care in assessing AVF maturation, currently standard ultrasound methods are limited to a 2-dimensional view of the geometry and thus do not allow for a true-patient specific hemodynamic analysis. Furthermore, ultrasound-derived flow rate relies on the Poiseuille flow theory of laminar flow in straight circular tubes, which do not describe the disturbed AVF flow well (47). However, more advanced ultrasound techniques have been developed (48). Furthermore, MRI-based CFD can provide additional information, such as wall shear stress, oscillatory shear index, and vorticity, which may help the clinician know how best to proceed to improve AVF maturation outlook and may help researchers design animal or cell experiments to delineate the molecular mechanisms and develop treatments. In our single-center, small study, the no-intervention group had a significantly larger WSS, and vorticity at 1 day following AVF creation. Larger and multi-center cohort studies with sufficient power are needed to validate this observation. Should this hypothesis hold true, CFD has the potential to be used as a clinical tool to help diagnose poor AVF remodeling at 1 or 2 days after creation vs. the current standard ultrasound at 6 weeks or longer. Additionally with recent advances in technology (e.g., high performance computers) MRI based CFD can now be fully completed within 1 day, making it a reasonable tool to add to the clinic.

Limitations of the study include this study’s MRI spatial resolution which did not allow visualization of hyperplasia. The luminal area results from the combination of lumen expansion and intimal hyperplasia, which decreases lumen area. To gain mechanistic insight into AVF remodeling, future studies can use imaging modalities with higher spatial resolutions and better soft-tissue contrast allowing for visualization of the blood vessel wall, especially between the adventitia and its adjacent, perivascular tissues. Additionally, MRI protocols used in the present study are prone to flow artifacts or reduced signals in the presence of stenosis or turbulent flow (47, 49, 50). Development of novel imaging modalities or methods such as renal disease patient-friendly contrast agents for MRI or computed tomography angiography could also lead to improved vascular imaging quality in these patients. Second, this study is limited to the juxta anastomosis region. The juxta anastomotic region was chosen due to its propensity to develop stenosis (51, 52). Future studies can consider a longer AVF segment to include other locations prone to stenosis, such as the cephalic arch. Third, our CFD analysis used a rigid blood vessel wall assumption. Future studies can consider a deformable blood vessel wall, i.e., fluid-structure interaction (FSI). However, FSI simulations may be less suitable to current clinical applications due to the required knowledge of the blood vessels *in vivo* biomechanical properties and wall thickness, which is challenging to obtain, and higher computational resources required. Additionally, blood-pressure measurements of AVFs could either be prescribed as part of the CFD set up or used as validation of the methods. Fourth, as an exploratory study, this study has 3 patients per group. Future studies can increase the patient number, consider confounding factors, and delineate associations between hemodynamics and AVF intervention.

## Conclusion

Our study reported temporal changes in luminal area and hemodynamic parameters in AVFs that did not require vs. required intervention before successful hemodialysis. Elucidating the differences between these two groups is essential to understanding the mechanisms of AVF remodeling, which in turn, could lead to developing therapies for promoting AVF maturation as well as improving diagnostic tools.

## Data availability statement

The datasets presented in this article are not readily available because the datasets presented in this article will be made available by the authors to the requester, after establishing a Data Usage Agreement between the requester’s institute(s) and the University of Utah. Requests to access the datasets should be directed to Y-TS, y.shiu@hsc.utah.edu.

## Ethics statement

The studies involving human participants were reviewed and approved by The University of Utah Institutional Review Board. The patients/participants provided their written informed consent to participate in this study.

## Author contributions

HN: data curation and formal analysis. HN, YH, and Y-TS: study conceptualization, methodology, software, investigation, writing – original draft, and writing – review and editing. HL, SB, and AKC: writing – review and editing. SB, AKC, and Y-TS: funding acquisition, project administration, and resources. Y-TS: responsible for supervision. All authors were responsible for methodology.

## Footnotes

1
www.vmtk.org
