# Supplementary material for: Differential hemodynamics between arteriovenous fistulas with or without intervention before successful use
Source: Front Cardiovasc Med. 2022 Nov 3;9:1001267. doi: 10.3389/fcvm.2022.1001267 (PMC9669082; doi:10.3389/fcvm.2022.1001267)
Supplement: Supplementary file 1 [file Image_1.pdf]

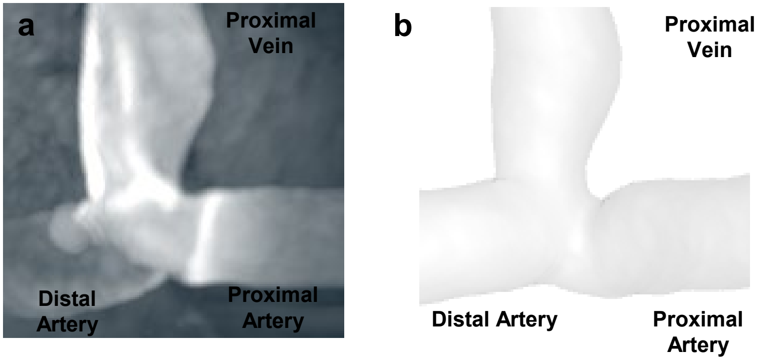

Supplemental Figure 1: Representative images showing (a) time of flight maximum intensity projection used to qualitatively verify accuracy of (b) 3D reconstruction of lumen.

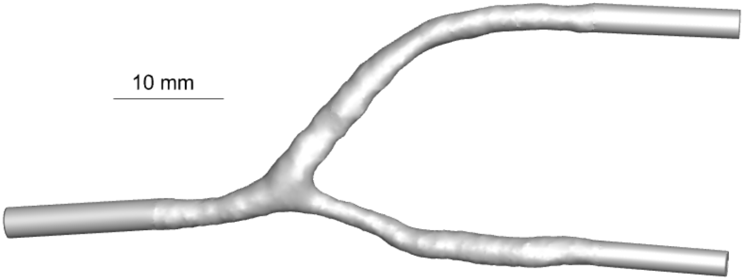

Supplemental Figure 2: Representative images of 3D lumen reconstruction with flow extensions.

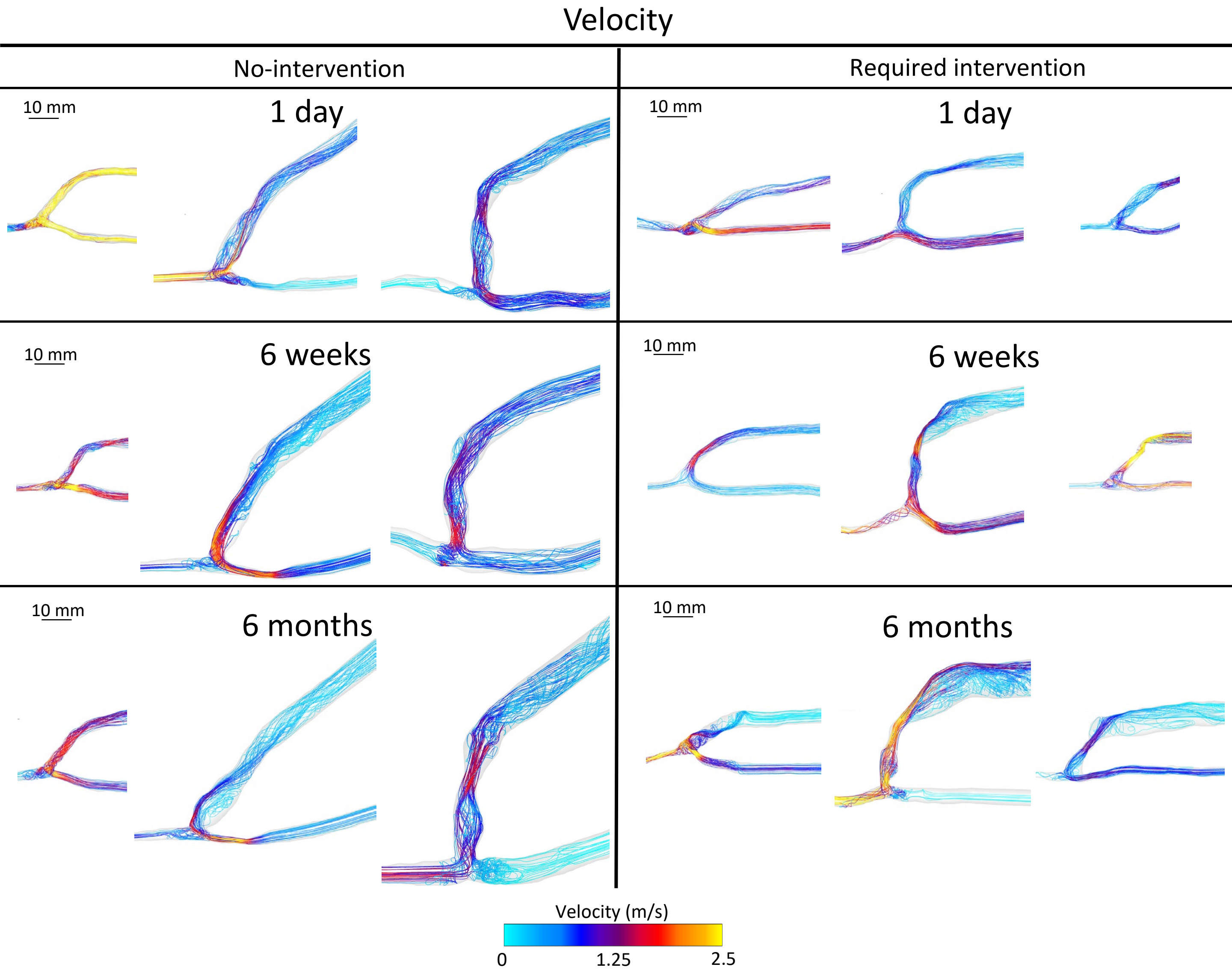

Supplemental Figure 3: Velocity streamlines of each AVF at systole.

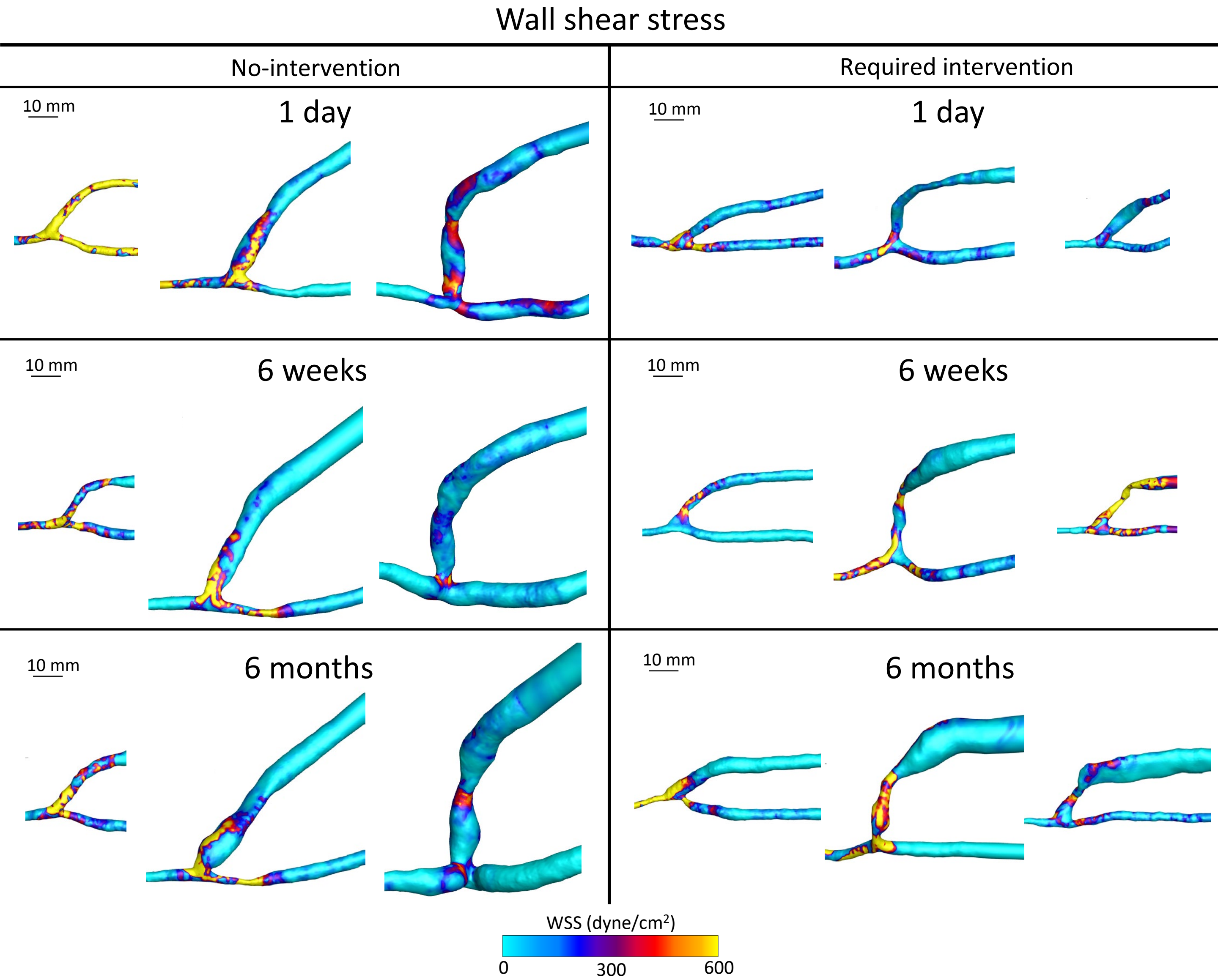

Supplemental Figure 4: Wall shear stress colormaps of each AVF at systole.

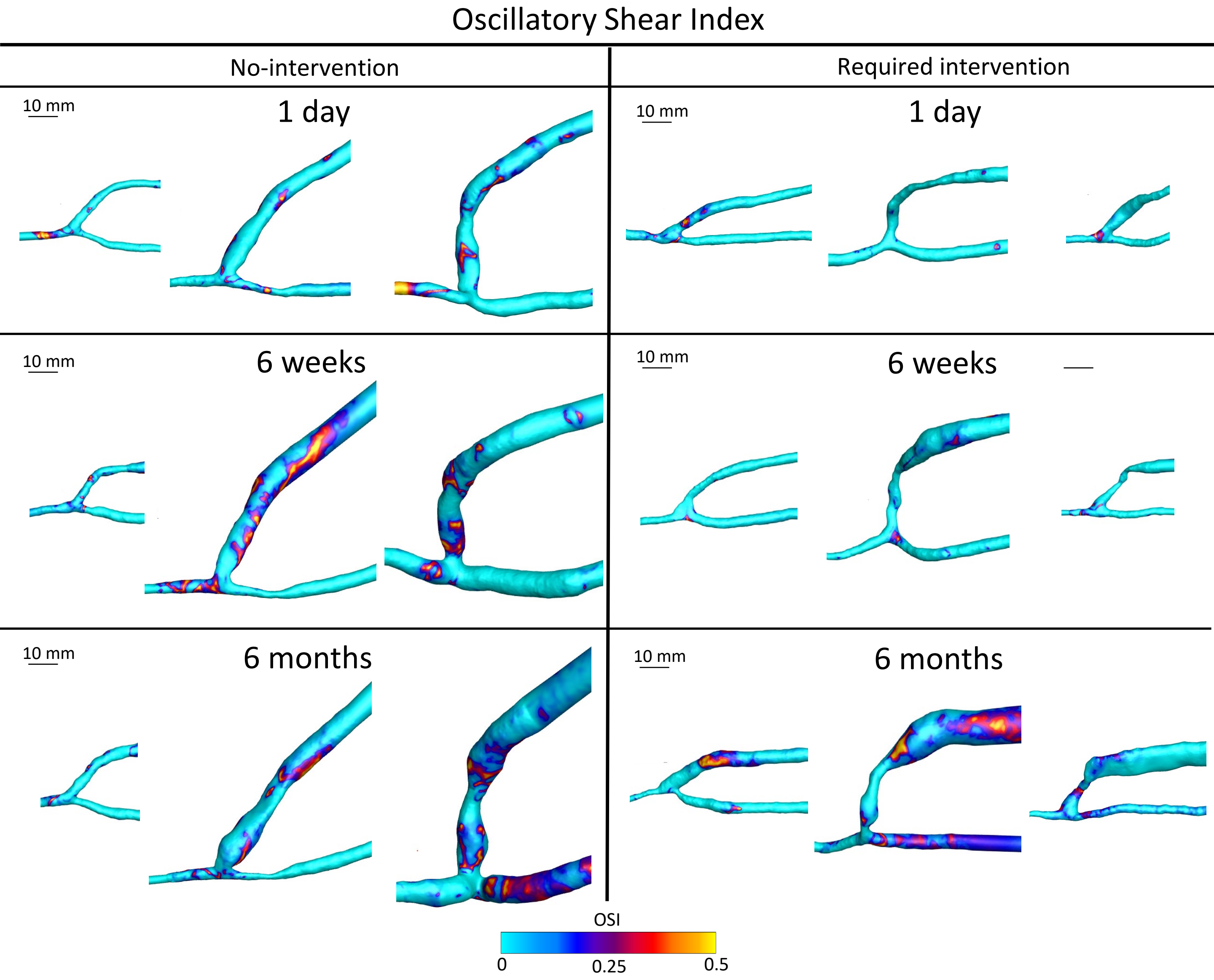

Supplemental Figure 5: Oscillatory shear index color maps of each AVF at systole.

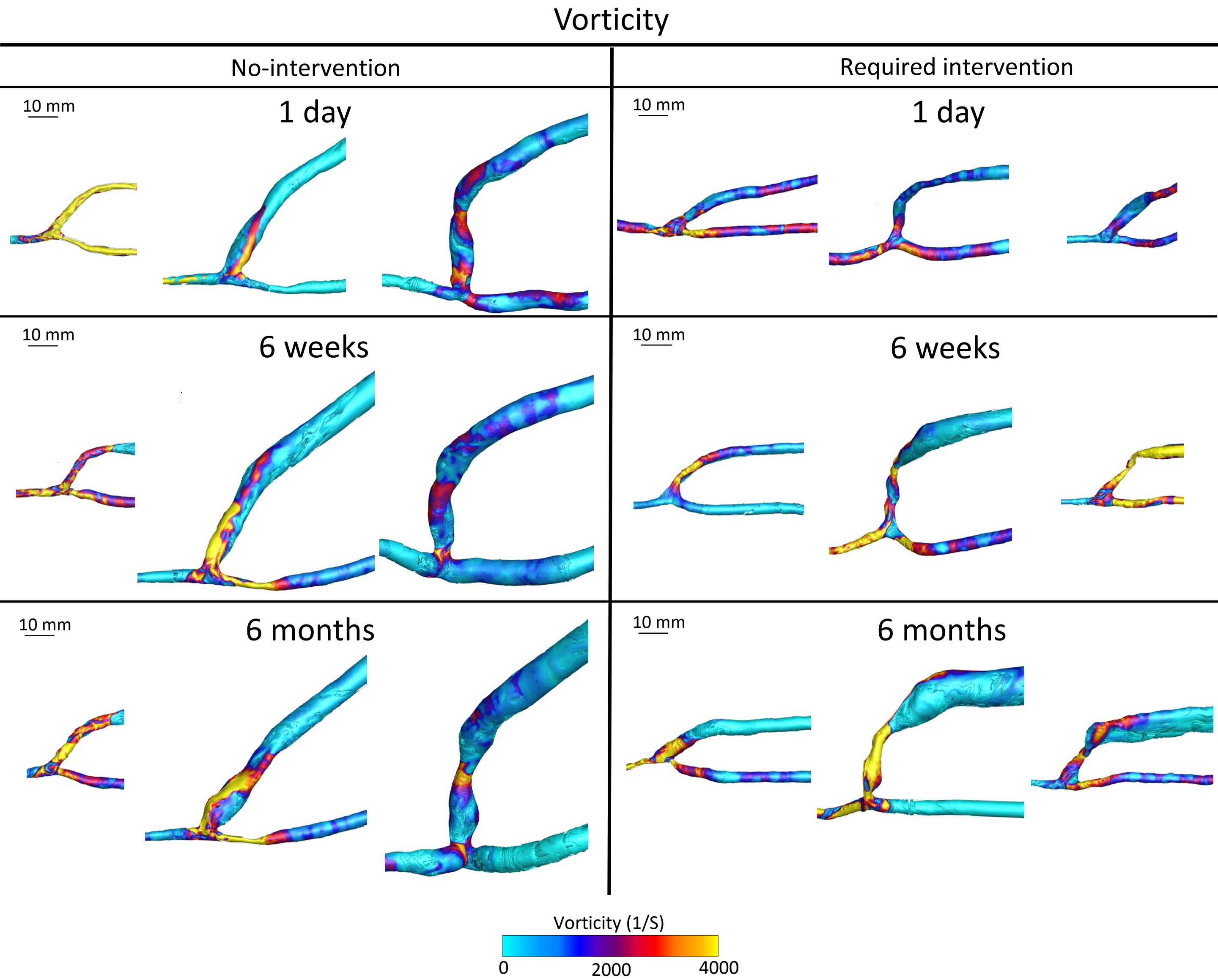

Supplemental Figure 6: Vorticity color maps of each AVF at systole.

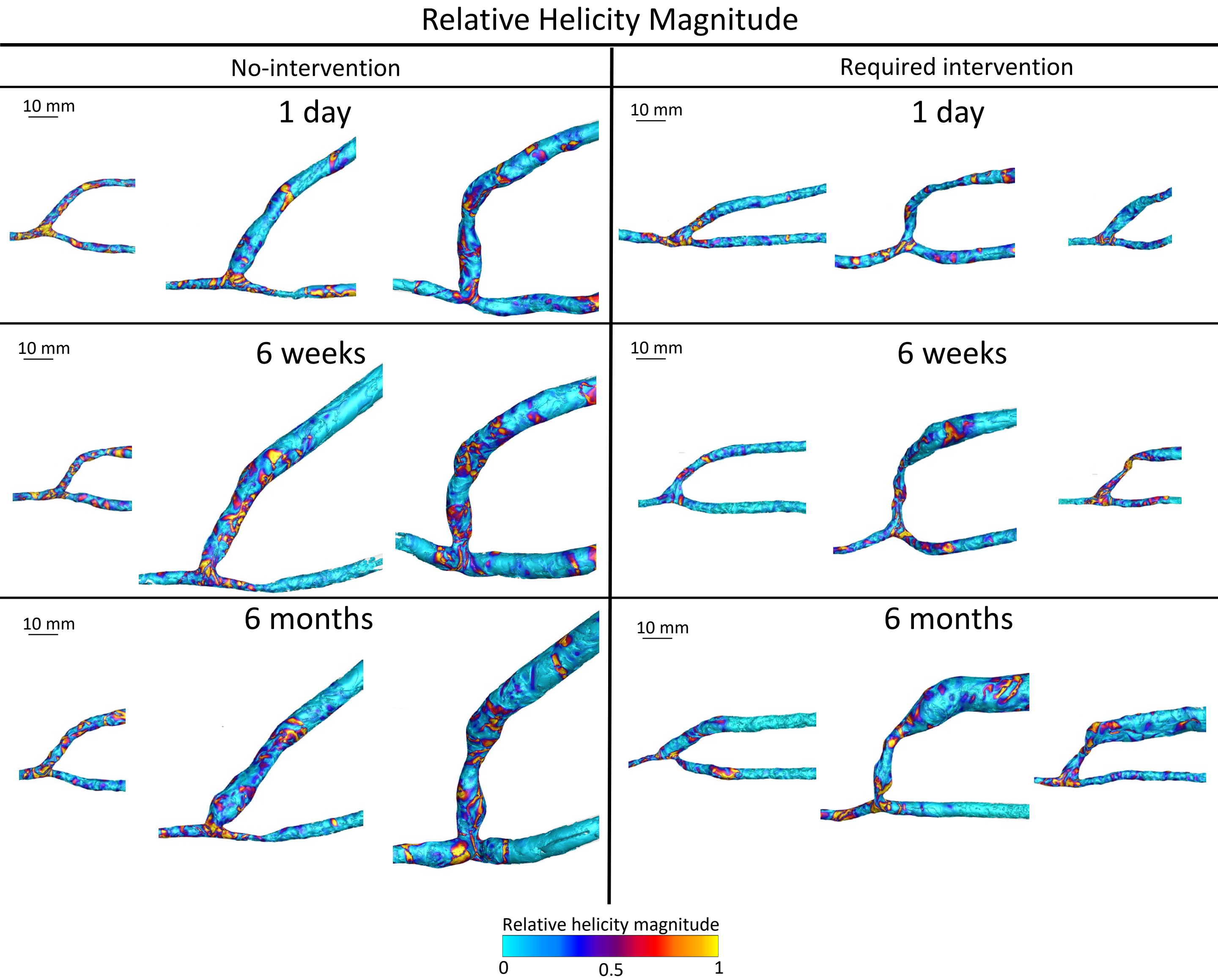

Supplemental Figure 7: Relative helicity magnitude color maps of each AVF at systole.
